# Theta-Defensins Inhibit High-Risk Human Papillomavirus Infection Through Charge-Driven Capsid Clustering

**DOI:** 10.3389/fimmu.2020.561843

**Published:** 2020-09-25

**Authors:** Joseph G. Skeate, Wouter H. Segerink, Mauricio D. Garcia, Daniel J. Fernandez, Ruben Prins, Kim P. Lühen, Féline O. Voss, Diane M. Da Silva, W. Martin Kast

**Affiliations:** ^1^Department of Molecular Microbiology & Immunology, Keck School of Medicine, University of Southern California, Los Angeles, CA, United States; ^2^Norris Comprehensive Cancer Center, University of Southern California, Los Angeles, CA, United States; ^3^Department of Obstetrics and Gynecology, Keck School of Medicine, University of Southern California, Los Angeles, CA, United States

**Keywords:** human papillomavirus, alpha-defensins, theta-defensins, infection, innate-immunology, sexually transmitted infection (STI)

## Abstract

Persistent infection with high-risk human papillomavirus (hrHPV) genotypes results in a large number of anogenital and head and neck cancers worldwide. Although prophylactic vaccination coverage has improved, there remains a need to develop methods that inhibit viral transmission toward preventing the spread of HPV-driven disease. Defensins are a class of innate immune effector peptides that function to protect hosts from infection by pathogens such as viruses and bacteria. Previous work utilizing α and β defensins from humans has demonstrated that the α-defensin HD5 is effective at inhibiting the most common high-risk genotype, HPV16. A third class of defensin that has yet to be explored are θ-defensins: small, 18-amino acid cyclic peptides found in old-world monkeys whose unique structure makes them both highly cationic and resistant to degradation. Here we show that the prototype θ-defensin, rhesus theta defensin 1, inhibits hrHPV infection through a mechanism involving capsid clustering that inhibits virions from binding to cell surface receptor complexes.

## Introduction

Human papillomavirus (HPV) remains the most common sexually transmitted infection within the human population and it is estimated that over 80% of all sexually active individuals will be infected at one point in their lifetimes (1). Persistent infection with a high-risk HPV (hrHPV) genotype is causally associated with the development of both anogenital and oropharyngeal cancers (2, 3). Even though prophylactic HPV vaccination has increased over the years, the United States still had an estimated 13,170 new cervical cancer cases diagnosed and ~4,250 deaths due to disease in 2019 (4). Additionally, the number of hrHPV-positive head and neck cancers occurring in men has seen a dramatic increase, underscoring that infection with hrHPV is a health burden on all sexually active individuals (5, 6). In both cases, the primary genotype associated with either cervical cancer (CC) or oropharyngeal squamous cell carcinoma (OPSCC) was HPV16, which is found in >50% of CC and >90% OPSCC (7, 8). As such, there is a continuous need to develop and test novel methods that may be used to prevent the transmission of hrHPV that can work alongside prophylactic vaccination and protect at-risk populations. A potential candidate to fill this need may be a unique member of antimicrobial peptides known as defensins.

Defensins are small cationic peptides of the innate immune system that are rich in cysteine and arginine residues (9). Originally studied because of their antibacterial activity, defensins have exhibited antiviral activity against both enveloped and non-enveloped viruses through interactions with both cellular receptors, viral anchoring proteins, direct binding to viral capsids, cell membrane fusion inhibition, and direct modulation of host responses to infection [for full review see (10, 11)]. While genetically similar, defensins are classified into α, β, and θ subgroups based on the three disulfide bond pairings and amino acid sequence similarities shared within each group (12). Uniquely, the θ-defensin family is the only known group of cyclic polypeptides expressed in the animal kingdom and exclusively found in old-world monkeys (13). Of the six different isoforms found in rhesus macaques, the Rhesus Theta Defensin-1 (RTD-1) isoform is the most abundant and accounts for over 50% of RTD content within azurophilic granules of neutrophils and can be detected in myeloid tissue (14). While humans also have genes that code for θ-defensins (termed retrocyclins), a premature stop codon within the sequences prevents their translation (15, 16). Structurally, this class of defensin is formed by the excision and binary ligation of two non-apeptides that create the mature 18-mer cyclic peptide (17, 18). This highly-cationic moiety is further stabilized by a core of three disulfide bonds, making it highly resistant to proteolytic cleavage and stable in blood, plasma, and serum (19).

In the context of hrHPV biology, the anti-viral efficacy of both α and β defensins have been explored. Buck *et al*. screened six different naturally produced defensins for their ability to inhibit HPV infection and found that β defensins showed minimal anti-viral activity while α-defensins, specifically HD5, were highly effective in blocking mucosal targeting hrHPV genotypes *in vitro* (20). Similar to α-defensins, θ-defensins have been shown to also exhibit anti-viral activities against several human pathogens including herpes simplex virus (HSV), human immunodeficiency virus (HIV-1), and influenza A virus (IAV), however have not been screened for efficacy against hrHPV (21–23). Given this, the purpose of our study was to examine whether the θ-defensin RTD-1 could inhibit prominent hrHPV genotypes 16, 18, and 31, and then characterize whether this inhibition was through viral capsid or host cell interactions.

## Materials and Methods

### Cell Lines

The cervical cancer cell line HeLa (CCL-2, ATCC) was maintained in Iscove's Modified Dulbecco's Medium (IMDM) supplemented with 10% heat-inactivated fetal bovine serum (FBS) (Omega Scientific, Tarzan, CA) and gentamycin (Lonza, Walkersville, MD). HaCaT cells, spontaneously immortalized keratinocytes, were cultured in Dulbecco's Modification of Eagle's Medium (DMEM) with 4.5 g/L glucose, L-glutamine, and sodium pyruvate (Corning, 10-013CV, NY) supplemented with 10% FBS (Omega Scientific), and gentamycin (Lonza). 293TT and 293TTF cells (kind gifts from John Schiller (NIH) and Richard Roden [Johns Hopkins University), respectively] were maintained in IMDM supplemented with 10% FBS (Omega Scientific) and gentamycin (Lonza). Episomal plasmids coding for additional SV40 large T antigen (293TT and 293TTF) and furin (293TTF) were maintained by the inclusion of 250 μg/mL hygromycin B (ThermoFisher) and 1 μg/mL puromycin (MilliporeSigma). All cells were grown in a humidified incubator at 37°C with 5% CO_2_ and passaged when confluency was approximately 80%.

### Antibodies

The HPV16 L1 antibody H16.V5, HPV18 L1 antibody H18.G10, and HPV31 L1 antibody H31.A6, used as neutralization controls within hrHPV infection studies, were gifts from Neil Christensen (The Pennsylvania State University). For western blot analysis of HPV surface binding the HPV16 L1 mAb CAMVIR-1 (550840, BD Bioscience), β-actin (4970, Cell Signaling Technologies), goat-anti-mouse IRDye 800CW (925-322, LI-COR), and goat-anti-rabbit (H + L) Alexa Fluor 680 (A27042, Thermo Fisher) were used. Immunofluorescent imaging was carried out using the HPV16 L1 antibody 56E.L1 (a kind gift from Martin Sapp, Louisiana State University), early endosomal antigen 1 (EEA-1) (Ab109110, Abcam), rabbit isotype control (02-6102, Thermo Fisher), mouse isotype control (03001D, BD Biosciences), TRITC goat-anti-rabbit (ab6718, Abcam), and DyLight 488 goat anti-mouse (405310, Biolegend).

### HPV Pseudovirus (PsV) and Virus Like Particle (VLP) Production

HPV16, 18, and 31 PsVs were prepared as previously described with the ripcord modification (24). Briefly, 293TT cells were co-transfected with codon-optimized L1 and L2 plasmids for HPV16 (p16L1L2), HPV18 (p18L1L2), and HPV31 (p31L1L2), along with a pCIneoGFP reporter plasmid (all gifts from John Schiller). Following a 2-day production, PsVs were matured overnight and purified on an iodixanol gradient (OptiPrep, MilliporeSigma, Burlington, MA). For bulk PsV preparations, the self-packaging p16L1L2 plasmid was utilized and the same purification method used. Infectious titer was determined by flow cytometric analysis of green fluorescent protein (GFP) + 293TT cells at 48 h post-addition of serially diluted PsV stock and calculated as infectious units (IU)/ml. Non-reporter plasmid containing PsV preps were quantified via coomassie blue staining with known BSA concentrations. To remove heparin sulfate proteoglycans (HSPG) that co-purify attached to the virion surface, a PsV prep was subjected to a brief trypsin and heparinase treatment prior to the addition of soybean trypsin inhibitor and purification on the iodixanol gradient and is referenced as “HSPG-” within the manuscript. Furin pre-cleaved HPV16 (fpc) PsV were generated using 293TTF cells using the protocol outlined by Wang et al. (25). Assessment of L2 cleavage by furin was carried out by western blot.

HPV16 virus-like particles (VLP) were produced using a recombinant baculovirus expression system as previous described (26). Western blot analysis confirmed the presence of HPV L1 and L2, while intact particles were verified by an enzyme linked immunosorbant assay using conformationally-dependent L1 antibodies. Coomassie Blue staining was performed to determine the concentration of HPV L1 protein within VLP preparations.

### Production of Defensins

The hydrochloride salts of RTD-1, 2, 4, and HD5 were prepared as previously reported producing pure (>98%) material verified by analytical reverse-phase ultra-performance liquid chromatography and tandem mass spectrometry (13, 17). Sterile stock solutions of were dissolved in saline and stored at 4°C. Defensin stocks were a kind gift from Michael Selsted (University of Southern California).

### HPV PsV Infection Assays

Stock PsV were titrated for each genotype so that the multiplicity of infection (MOI) given for experiments resulted in ~30% gene transduction at 48 h post addition. HeLa or HaCaT cells were seeded at 2 × 10^4^ cells/well in 24-well plates and incubated overnight at 37°C prior to infection assay. PsV were diluted in IMDM without FBS and combined with indicated concentration of RTD-1, RTD-2, RTD-4, HD5, or neutralizing antibody in a total volume of 100 μl for 1 h at 37°C and then added to cells. Alternatively, cells were pre-incubated with indicated concentration of RTD-1 for 1 h at 37°C, excess, unbound RTD-1 was washed away with warm PBS, and HPV16 PsV added.

For dialysis experiments, RTD-1, HPV16 PsV, or RTD-1 + PsV combinations in IMDM with phenol red were loaded into Pierce 96-well Microdialysis cassettes (88260, Thermo Fisher) and dialyzed against 2 L of PBS in separate containers for 4 h at room temperature (RT) (4 × 500 mL exchanges, 1 per h). Dissociation of phenol red out of cassettes was used as a visual indicator of complete exchange. Post dialysis, PsV and RTD-1 + PsV were removed from cassettes and added to HeLa cells for 48 h. Separately dialyzed PsV and RTD-1 were combined for 1 h at 37°C before addition to cells and served as a control to show RTD-1 was dialyzed out of cassettes.

For all infection readouts, cells were trypsinized, collected, and analyzed on a Cytomics FC500 using CXP software (version 2.2) (Beckman Coulter). Infection was defined as percentage of GFP expressing cells within treatment groups. Viability was assessed concurrently through the use of a propidium iodide stain.

### HPV16-pHrodo VLP Uptake Assays

HPV16 VLP were labeled with pHrodo-red iFL (ThermoFisher) according to manufacturer's instructions at a dye to L1 protein ratio of 20:1. Excess dye was removed from VLP preps through agarose bead column filtration. The pHrodo labeled VLPs were re-quantified through coomassie blue staining with known BSA concentrations.

HeLa cells were seeded at 2.0 × 10^4^ cells/well in a 48-well plate and incubated overnight before uptake assays. pHrodo-labeled VLPs were mock treated or incubated with indicated concentrations of RTD-1, RTD-2, RTD-4, or HD5 for 1 h at 37°C. After treatment of VLP, media was removed from cells and replaced with VLP or VLP + defensin combination at a concentration of 1.25 μg L1/1 × 10^6^ cells. Cells were incubated at 37°C for duration of uptake assay with intensity of pHrodo-red signal read every hour for the duration of the experiment on a CLARIOstar Plus microplate reader (BMG Labtech).

### NanoSight Imaging of Viral Particles

HPV16 VLP were diluted in PBS or treated with indicated concentrations of RTD, HD5, or RTD+VLP combination in 100 mM CaCl_2_. After incubation, samples were run on a NanoSight NS300 (Malvern Panalytical) and particle size/number was quantified using Nanoparticle Tracking Analysis (NTA) software (version 3.2).

### Confocal Imaging and Colocalization Analysis

For HPV16 colocalization with EEA1, HeLa cells were seeded into 8-well chamber slides (ibidi) at 1 × 10^4^ cell/well and grown overnight. HPV16 PsV were diluted in PBS or treated with indicated concentration of RTD-1 or HD5 for 1 h at 37° prior to addition to cells. Cells were treated with 0.5 μg PsV/1 × 10^6^ cells for 2 h at 37 °C. After treatment, cells were gently washed with PBS to remove unbound excess virions and then fixed with 4% paraformaldehyde. Cells were permeabilized with triton X-100 and stained overnight in a humidified chamber at 4°C for HPV16 (H16.56E, 1:200) and EEA1 (1:250). After secondary antibody incubation for 30 min at RT, excess antibody was removed, and slides were coverslip mounted with ProLong Gold Antifade Mountant with DAPI (Thermo Fisher). Immunofluorescent (IF) images were captured on a Nikon Eclipse Ti-E laser scanning confocal microscope running Nikon Elements software (version 4.0). For colocalization analysis, 15 images from each treatment group with >20 cells/image were analyzed with Fiji (a distribution of ImageJ, NIH) (27). Using the JACoP plugin thresholds were automatically set, and the extent of colocalization was measured and reported as Mander's colocalization coefficient (28).

### Transmission Electron Microscopy Imaging

HPV16 PsV were diluted in PBS or treated with indicated concentration of RTD-1 for 1 h at 37° prior to mounting on Formvar-coated copper support grids (FF200-Cu, Electron Microscopy Sciences). Grids were washed by floating on sterile PBS followed by counterstaining with uranyl acetate (22,400, Electron Microscopy Sciences). Images of VLP were collected on a JEOL JEM-2100 LaB6 electron microscope running Gatan imaging software (JEOL Ltd., Akishima, Tokyo).

### Western Blot Analysis of Binding

HeLa cells were seeded at 5.0 × 10^5^ cells/well in 6-well plates and incubated overnight at 37°C prior to binding assay. VLP were diluted in IMDM without FBS and combined with indicated concentrations of RTD-1, HPV16.V5 antibody, or PBS for 1h at 37°C. Tissue culture plates were cooled to 4°C, media was removed, and cells were gently washed with cold PBS. VLP or treated VLP were then added to cells and bound for 1 h at 4°C followed by removal of excess unbound virions through two washes with PBS. Cells were then collected through scraping and membrane fraction isolated with Mem-PER reagent (89842, Thermo Fisher) using the manufacturer protocol. Protein concentrations were quantified between samples through a Bradford assay and 20 μg of cellular protein was run on a NuPAGE 10% Bis-Tris gel (Thermo Fisher) in MOPS buffer. Gels were transferred to nitrocellulose using the iBlot2 system (Thermo Fisher), blocked for 1 h in StartingBlock buffer, and then stained overnight at 4°C for HPV16 L1 (CAMVIR, 1:2,500) and β-actin (CST, 1:1,000). The following day blots were washed, secondary antibodies were added for 45 min at RT, then blots were imaged on an Odyssey imaging system (LI-COR). Band intensity within images were then quantified using Image Studio Lite software (version 5.2.3, LI-COR).

### Statistical Analysis

For this study analysis was performed using GraphPad Prism (version 8.4.2, GraphPad Software) with a minimal threshold set at *p* ≤ 0.05 for significance. Details for statistical analysis for each individual experiment can be found within figure legends.

## Results

### Pretreatment of hrHPV Pseudovirions (PsV), Not Cells, With RTD-1 Results in Dose-Dependent Inhibition of Infection

**Figure 1:**
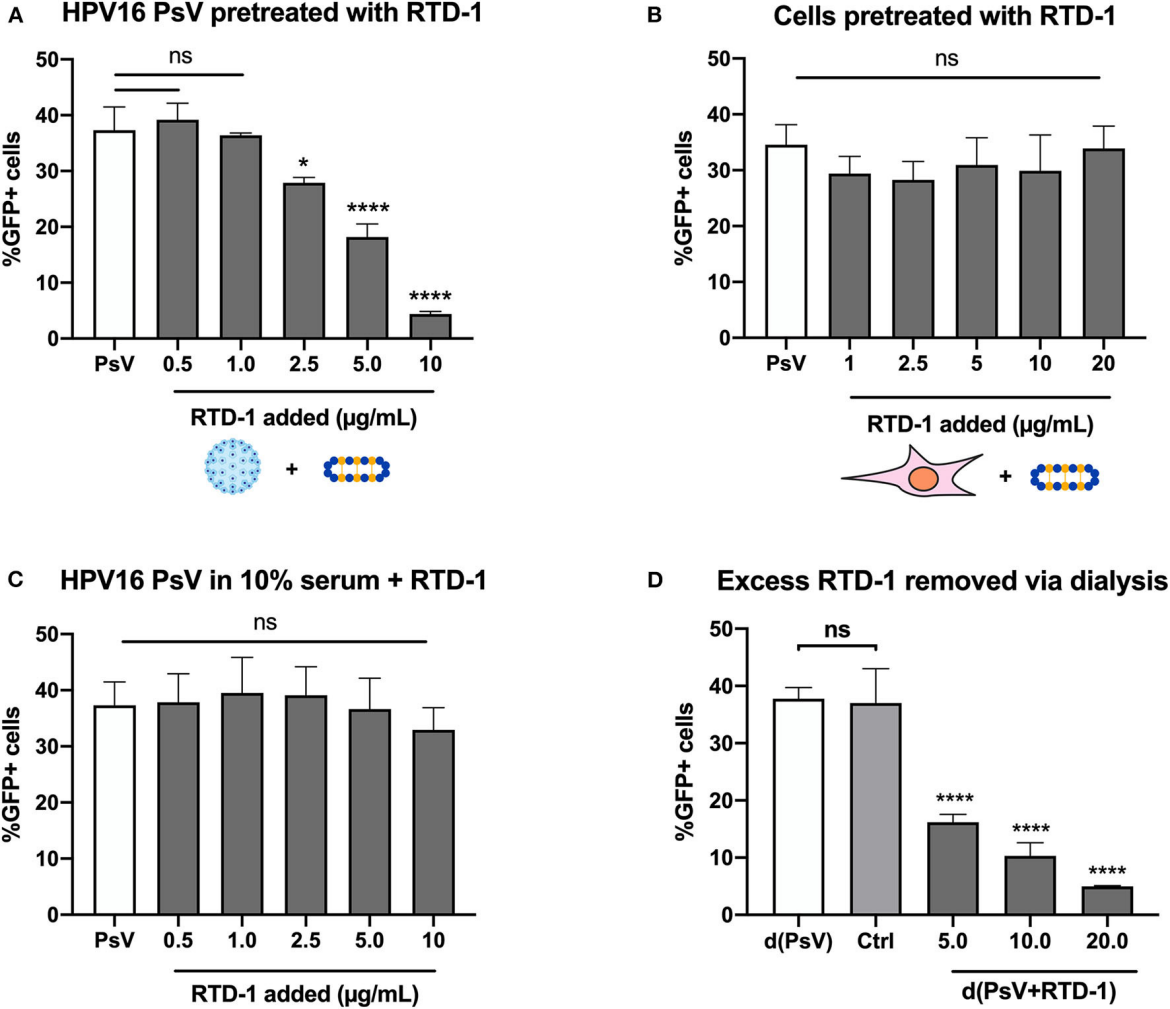
RTD-1 inhibits HPV16 PsV infection in a dose-dependent manner driven by an interaction between viral capsid and θ-defensin. **(A)** HPV16 PsV pre-incubated with RTD-1 in serum-free medium, **(B)** HeLa cells pre-incubated with RTD-1, or **(C)** HPV16 PsV pre-incubated with RTD-1 in the presence of serum were added to cells for 48 h prior to measurement of GFP expression resulting from PsV gene transduction. **(D)** Alternatively, excess RTD-1 was dialyzed out of PsV+RTD-1 (d(PsV + RTD-1) combinations, with separately dialyzed PsV [d(PsV)] combined with dialyzed RTD-1 (20 μg/ml) to indicate removal of RTD-1 from dialysis cassettes (Ctrl). Each panel shows the percentage of GFP+ (PsV infected) cells ± SD as analyzed by flow cytometry. All experiments were carried out in triplicate. Results shown are representative data from 3 independent experiments (ns = not significant, ^*^p < 0.05, ^****^p < 0.0001, one-way ANOVA followed by Dunnett's multiple comparisons test against PsV within groups).

HPV16 pseudovirions (PsV) were used to examine whether the θ-defensin RTD-1 could inhibit infection, as measured by gene transduction of the reporter plasmid pCIneoGFP within HPV PsV, resulting in GFP expression by target cells. Initial screening involved using serial dilutions of RTD-1 in serum-free IMDM and combining them with PsV for 1 h prior to addition to cells. 48 h post infection GFP + cells were assessed by flow cytometry. Through this method we were able to clearly see a dose-dependent inhibition on infection rates with significant reductions starting at 2.5 μg/ml of RTD-1 (Figure 1A). To address whether the inhibition was resultant of RTD-1 interacting with cell surface proteins or virions, we used several approaches. First, we found that pretreating cells directly for 1 h with increasing concentrations of RTD-1 prior to PsV addition resulted in no significant change in infection levels (Figure 1B). Next, we found that the presence of serum during the PsV+RTD-1 pre-incubation steps resulted in a failure of RTD-1 to inhibit PsV infection (Figure 1C), suggesting that competing serum proteins can interfere with the initial interaction between RTD-1 and the virus capsid. It is worth noting that this “serum effect” has also been documented in other defensin studies and was used as an indirect way to show defensin-pathogen interactions (20, 29, 30).

To investigate whether “free” RTD-1 in the PsV + RTD-1 mixture contributed to inhibition of HPV16 infection, we used dialysis as a method to take advantage of the size discrepancies between RTD-1 (~2 kDa) and PsVs (>20 megaDa) (31). After the initial 1 h pre-incubation of virions and RTD-1 we used a large molecular weight membrane to dialyze out unbound RTD-1 prior to PsV collection and addition to cells. Post dialysis the PsV + RTD-1 combinations were collected, diluted in IMDM containing 10% FBS, and added to cells for the infection assay. Separately dialyzed PsVs were used as a control to verify there were no effects of the dialysis procedure on baseline infectivity. To provide evidence that excess RTD-1 passes through the membrane, we dialyzed the highest concentration of RTD-1 (20 μg/ml) in a separate container in the same manner, collected the dialyzed solution, and combined it with the dialyzed PsV for 1 h in serum-free IMDM before adding it to the cells in complete media for the infection assay (labeled as Ctrl) (Supplementary Figure 1). Significant, dose-dependent decreases in the PsV infection rate were seen in the PsV + RTD-1 combinations while there was no change in infection rate with the control dialyzed RTD-1 combined with PsVs (Figure 1D). Taken together, this set of data suggests that the infection inhibition occurs through interactions between the PsV and RTD-1 directly and not through binding of RTD-1 to cell surface virus entry receptors.

### RTD-1 Significantly Reduces Uptake of HPV16 by Inhibiting Initial Binding of Defensin-Coated Virions to The Cell Surface

**Figure 2:**
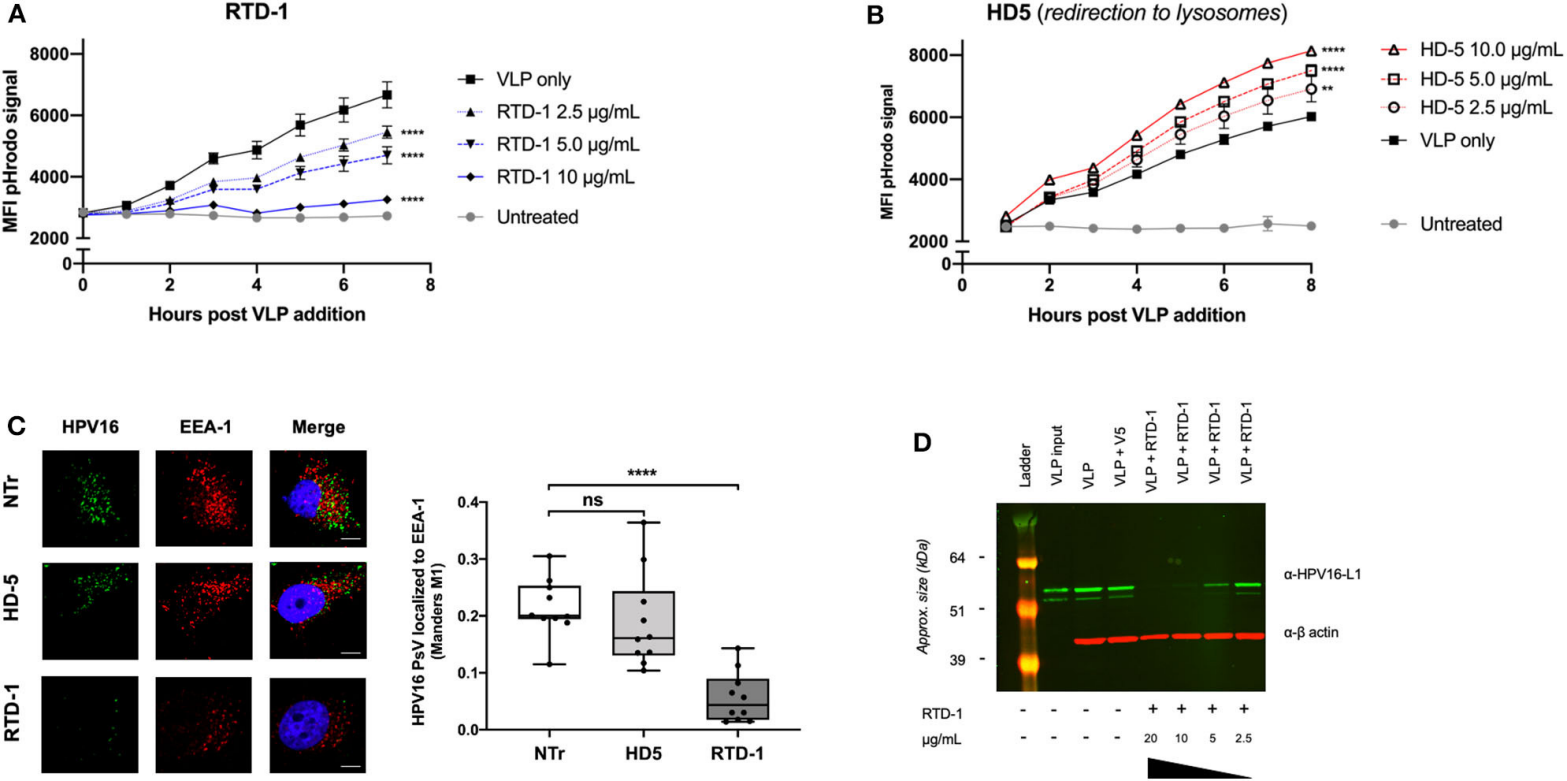
RTD-1 treatment of HPV16 results in significantly reduced uptake and localization of virions to early endosomes highlighted by sparse detection of large aggregates at cell surfaces and reduced cell binding capacity of RTD-1 treated virions. Time course of pHrodo-HPV16 VLP internalization and trafficking to low pH endosomal compartments in the presence of **(A)** RTD-1 or **(B)** HD5. Shown is the mean pHrodo signal intensity of triplicate wells ± SD. Background signal from untreated cells included. **(C)** Immunofluorescence imaging of internalized HPV16 PsV untreated (NTr) or incubated with 5.0 μg/ml RTD-1 or HD5. (Left) Representative images from each field of view per treatment group (scale bar = 10 μm). (Right) Quantitation of HPV16 and EEA-1 colocalization was performed using ImageJ with the JaCOP plugin using 10 individual fields of view (10–20 cells/field) from two independent experiments. **(D)** Western blot of cell surface bound HPV16 L1 capsid protein in the presence of RTD-1. Membrane fractions are shown, probed for HPV16 L1 (green) and actin (red). ns = not significant, ^**^p < 0.01, ^****^p < 0.0001 two-way ANOVA followed by Dunnett's multiple comparison test against untreated VLPs at each timepoint for uptake experiments. One-way ANOVA followed by Dunnett's multiple comparison test against untreated PsV in colocalization studies.

Evidence suggests that the α-defensin HD5 inhibits HPV infection through direct capsid interactions that prevent viral uncoating, which redirects the trafficking of virions to lysosomes for degradation (32, 33). Because our data similarly suggested a direct interaction between RTD-1 and the viral capsid, we wanted to examine the effects of HPV uptake using HD5 inhibition as a comparison. As we have previously reported, pHrodo-labeled HPV16 virus like particles (VLPs) can be used to evaluate uptake as the fluorescence intensity of the label increases as particles are trafficked to successively lower pH endosomal compartments (34, 35). After pre-incubating the pHrodo-labeled VLP with increasing concentrations of RTD-1 or HD5, complexes were added to HeLa cells and hourly changes in fluorescence intensity were measured to assess viral uptake. We found that pre-incubating HPV16 pHrodo-labeled VLP with 2.5 μg/ml of RTD-1 resulted in significant decreases in pHrodo-particle signal beginning at 3 h post addition, and that the higher concentrations of RTD-1 reduced the pHrodo-particle signal to near background levels, suggesting an elimination of viral uptake (Figure 2A). In contrast, pretreatment of pHrodo-labeled VLP with HD5 resulted in significant increases in pHrodo-VLP signal throughout the time course (Figure 2B), consistent with augmented redirection of virus particle to acidic compartments. These findings are in support of the previous study by Wiens et al., who demonstrated that HD5 stabilizes the capsid and redirects it to the lysosome (33).

Given that pHrodo-labeled VLP signal is not a direct quantitative measure of uptake but rather a combination of the quantity of particles and their endosomal location, we next sought to examine whether there were changes in the localization of HPV virions to early endosomal compartments. Using immunofluorescence imaging we found that, after 3 h of uptake, there was no significant difference between the number of untreated and HD5-treated HPV16 PsV localized to EEA1 + early endosomes, however RTD-1-treated virions had a significant reduction in colocalization events (Figure 2C). Interestingly, there were very few cells that had positive signal for HPV16 when PsV were pre-incubated with RTD-1, however the ones that did, showed large, irregular areas of bright staining (Supplementary Figure 2). These data suggest that RTD-1 may be driving viral particles to cluster/aggregate and stay at the surface of specific cells while failing to bind to others.

Since our pHrodo data indicated a limited amount of virus particle uptake (confirmed by colocalization analysis), we decided to examine whether RTD-1-treatment of virions reduced their capacity to bind to the cell surface, which is a requisite step to initiate infectious viral entry (36–38). After initial pretreatment of HPV16 VLP with increasing concentrations of RTD-1, virions were bound to pre-cooled HeLa cells for 1 h at 4°C. Excess, unbound virions were gently removed with cold PBS washes and cells were harvested via scraping into separate 1.5 mL siliconized tubes. Membrane and cytosolic factions were extracted to quantify the relative amounts of virions bound to the cell surface between groups. Western blot of membrane fractions clearly indicated a dose-dependent decrease in the amount of L1 capsid protein recovered from the cell surface (Figure 2D). This indicates that as the concentration of RTD-1 treatment increases, the ability of virions to effectively bind to the cell surface is lost.

### RTD-1 Interacts With the Viral Capsid and Causes Capsid Clustering

**Figure 3:**
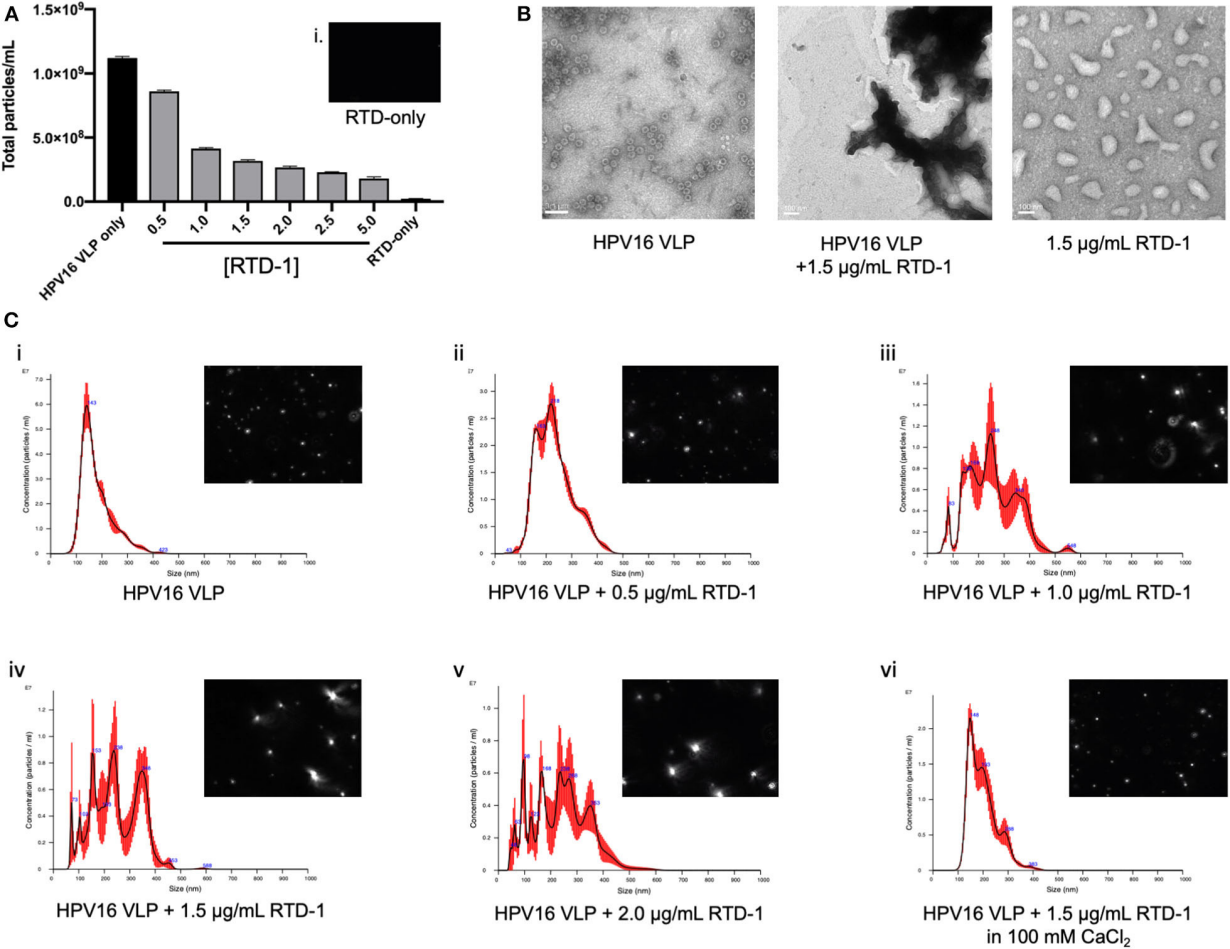
RTD-1 interacts with HPV16 VLP to form viral aggregates and can be prevented with the addition of excess divalent cations during incubation. HPV16 VLP were incubated with RTD-1 at indicated concentrations and run on a NanoSight NS300 to quantify number of particles or mounted on formvar-coated copper grids and imaged via TEM. **(A)** Shown is the mean quantification of particles/ml as analyzed by nanoparticle tracking analysis (NTA) through five separate video segments (± SD), insert (i) shows background signal from RTD-1 without VLP present. **(B)** TEM imaging of HPV16 VLP, VLP incubated with 1.5 μg/ml RTD-1, or RTD-1 alone (scale bar = 100 nm). **(C)** Representative histograms generated by nanoparticle tracking analysis (NTA) showing mean particle sizes and quantity ± SEM (red bars). Inserts on each show a single frame from the videos the particle analysis was performed on.

Our data to this point provides evidence that RTD-1 is able to inhibit HPV16 infection through reducing uptake potentially driven by an inability of RTD-1 treated virions to interact with the cell surface, however it was not known whether the θ-defensin treatment of virions was disrupting individual capsid structures or causing viral aggregates. To examine this, we utilized nanoparticle tracking analysis (NTA) to assess whether the size and quantity of particles was being modified with RTD-1 treatment. With this light scattering method we noted that the overall number of particles that flowed through the NS300 flowcell were reduced as the concentration of RTD-1 was increased (Figure 3A). Concurrently, we mounted HPV16 VLP and RTD-1 treated VLP on formvar-treated grids and performed TEM imaging. Untreated VLP show the characteristic 55 nm virus structure whereas no individual virus particles could be seen in the presence of RTD-1 (Figure 3B). RTD-1 by itself did not form large negatively stained protein aggregates. Histograms of particle size based on quantity clearly showed a demarcated increase in larger particles as the concentration of RTD-1 increased (Figures 3Ci–v). This quantified increase in particle size can also be clearly visualized from the individual frames from videos taken with the NS300 (histogram inserts). Together, these three observations suggest that viral particles are aggregating in the presence of RTD-1. Finally, in an effort to establish whether these capsid clusters are forming due to the highly cationic nature of RTD-1, we added in an excess of divalent cations (100 mM CaCl_2_) to the PsV+RTD-1 mixture and observed that the histogram distribution of particle sizes nearly returned to non-RTD-1 treated samples (Figure 3Cvi). These findings suggest that by saturating the environment with positive charged ions RTD-1 is unable to interact with the virions and induce aggregation. Videos from individual sample runs are included for visual reference of light scattering between VLP only, VLP + 1 μg/ml RTD-1, VLP + 2 μg/ml RTD-1, VLP + 5 μg/ml RTD-1, and RTD-1 alone (Supplementary Videos 1–5).

### RTD-Mediated Inhibition of HPV Infection Is Dependent on the Innate Charge of the θ-Defensin Isoform

**Figure 4:**
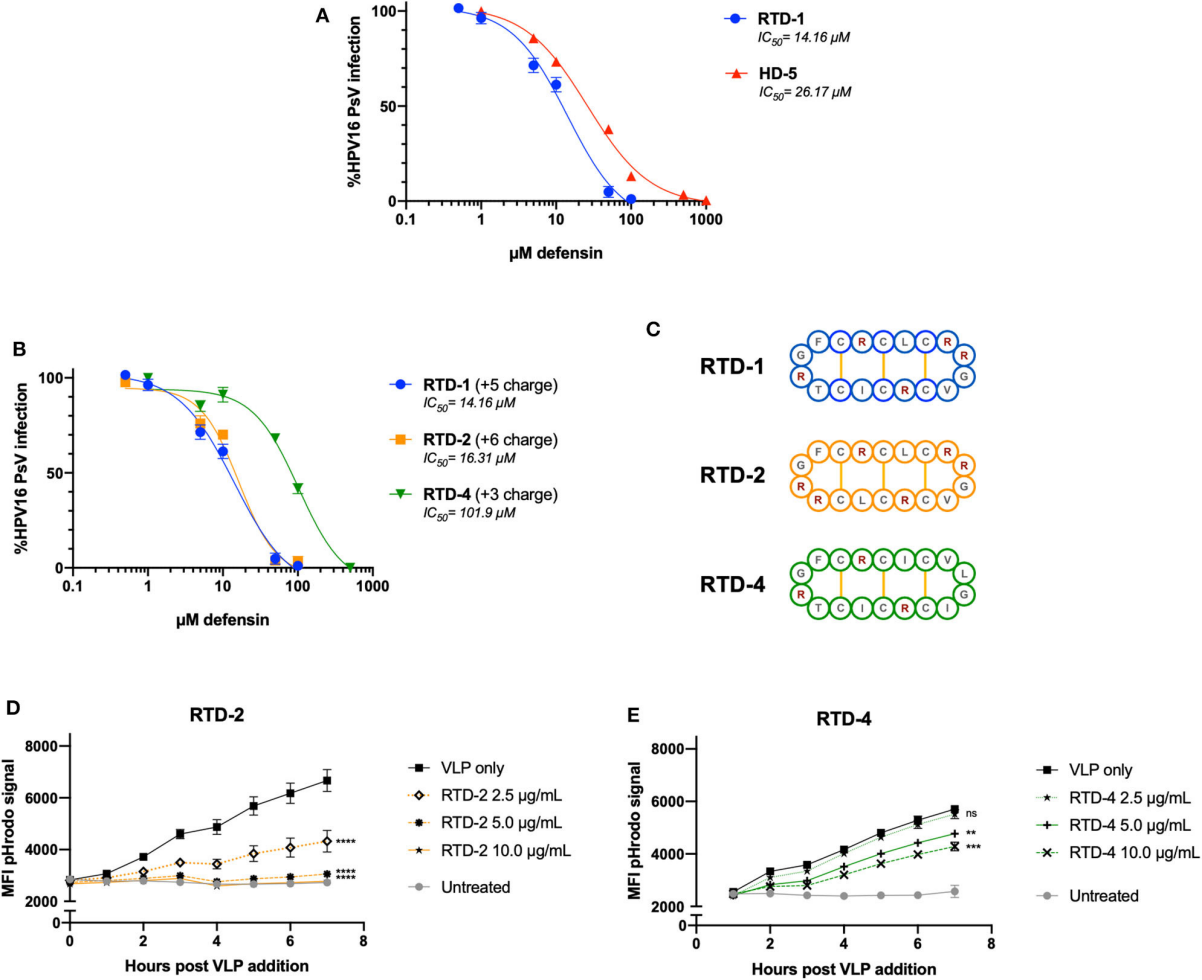
The viral inhibitory concentration of RTD-1 is similar to HD5 and is dependent on the charge of the RTD isoform. **(A,B)** HPV16 PsV were pretreated with indicated concentrations of HD-5, RTD-1, RTD-2, or RTD-4 prior to addition to HeLa cells. Infection rates were normalized to untreated PsV infection and inhibition curves generated for each defensin. Shown is a non-linear regression curve fit with IC50 values shown. Individual dots are the mean of triplicate infection rates values ± SD. **(C)** Cyclic amino acid sequence diagram of RTD-1, RTD-2, and RTD-4 with positively charged sidechains indicated in red and disulfide bridges in yellow. **(D,E)** pHrodo-labeled HPV16 VLPs were pretreated with indicated concentrations and added to HeLa cells. Mean fluorescent intensity of pHrodo signal was measured hourly to assess uptake and is shown as the average of three separate wells at each time point ± SD. All figures are representative of data from at least three independent experiments. ns = not significant, ^**^p < 0.01, ^***^p < 0.001, ^****^p < 0.0001 two-way ANOVA followed by Dunnett's multiple comparison test against untreated VLPs at each timepoint for uptake experiments.

After noting that excess positive charge within RTD-1 + VLP combinations inhibited the formation of aggregates seen in the nanoparticle tracking analysis, we wanted to confirm whether the inherent charge of the θ-defensin had an effect on infection inhibition and viral uptake. To test this, we compared RTD-1 (+5 charge) to two different RTD isoforms: RTD-2 (+6 charge) and RTD-4 (+3 charge) (Figure 4C). As a positive control for defensin-mediated inhibition of HPV infection we included HD5 in our experiments. Importantly, we found that RTD-1 and HD5 were both able to inhibit HPV16 infection within the μM-range with RTD-1 having an IC50 at approximately 14 μM while the HD5 IC50 was 26 μM (Figure 4A). RTD-4, with almost half the charge of RTD-1, had an IC50 of 101 μM, suggesting that reduced charge leads to reduced infection blocking (Figure 4B). This reduced capacity to inhibit infection was also seen in the HPV16 pHrodo-labeled VLP uptake assay as a greater concentration of RTD-4 was needed to significantly inhibit uptake in HeLa cells (Figure 4E). RTD-2 showed a nearly identical inhibitory IC50 to RTD-1 (Figure 4B) and showed a concentration-dependent effect on viral uptake (Figure 4D) which was much more effective than RTD-4 at matched concentrations. These results suggest that the inherent charge of the RTD-1 isoform dictates the ability of the θ-defensin to inhibit VLP uptake and HPV16 PsV infection.

### RTD-1 Is Able to Inhibit Other hrHPV Genotypes, Furin Pre-cleaved (fpc) HPV16 PsV, and Ability to Inhibit Is Influenced by The HSPG-Status of Virions

**Figure 5:**
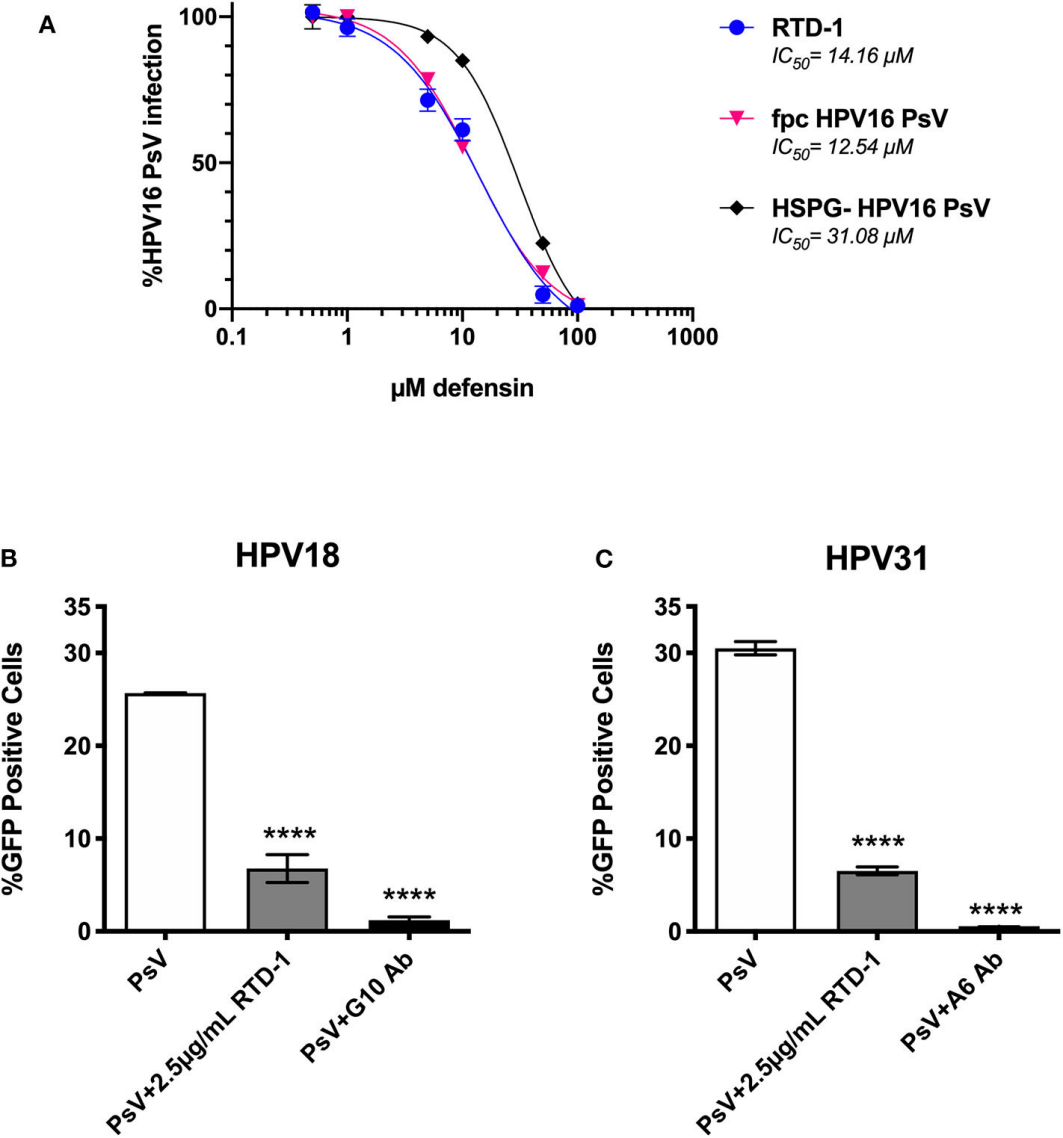
RTD-1 is able to inhibit furin pre-cleaved (fpc) HPV16 PsV, capacity to inhibit is partially influenced by the HSPG-status of virions, and RTD-1 has cross hrHPV genotype efficacy. Furin pre cleaved (fpc) HPV16 PsV, HPV16 PsV that had been treated with heparinase and trypsin before purification (HSPG-), and hrHPV genotypes 18 and 31 shown. **(A)** RTD-1 is able to inhibit fpc HPV16 PsV and has a reduced capacity to inhibit HSPG-free HPV16 PsV. Infection with non-treated HPV16 PsV, fpc PsV, or HSPG- PsV were used to normalize infection. Individual points on graph are means of triplicate wells ± SD with a non-linear regression curve. IC_50_ for each curve are shown. RTD-1 inhibits infection of HeLa cells by both **(B)** HPV18 and **(C)** HPV31 high-risk genotypes. Shown is the mean %GFP+ cells of triplicate wells ±SD. Genotype specific antibodies were used for neutralization controls. **(C)** ^****^p < 0.0001, one-way ANOVA followed by Dunnett's multiple comparisons test against PsV-only group.

Given that furin-processing at the cell surface is requisite for successful infection and partially dictates the asynchronous uptake of HPV virions (39, 40), we sought to assess whether RTD-1 would still be able to inhibit fpc HPV16 PsV. We found that fpcPsV are just as sensitive to RTD-1 inhibition as regular PsV (Figure 5A). Interestingly, removal of HSPG moieties that may co-purify during normal PsV production resulted in a PsV (HSPG-) that was slightly more resistant to RTD-1 inhibition as the IC_50_ was found to be roughly twice the amount needed to inhibit unmodified PsV (Figure 5A). Additionally, while HPV16 is the dominant genotype found in both cervical and head and neck cancers, we still wanted to examine the effects of RTD-1 on infection by other prevalent hrHPV to confirm a multi-hrHPV subtype effect (41). Using genotype-specific neutralizing antibodies as positive controls for inhibition, we found that RTD-1 was able to significantly reduce infection of HPV18 and HPV31 PsV in a dose-dependent manner, similar to HPV16 (Figures 5B,C, 2.5 μg/ml shown), suggesting that RTD-1 inhibition can be applied across many hrHPV genotypes.

Taken together, the overall results provide evidence that RTD-1 and other cyclic θ-defensin isoforms are able to inhibit hrHPV infection through charge-based capsid clustering and inhibition of infectious uptake in target cells.

## Discussion

Antimicrobial peptides play essential roles in defending epithelial tissues against infection by viruses and bacteria (12). Defensins are a subclass of these innate immune components and have been found in both plants and animals (42). Previous work has demonstrated that α defensins, specifically HD5, were most effective in inhibiting HPV infection out of all human-produced defensins screened (20). Within this study we have now expanded the list of defensins that act against HPV to include θ-defensins by characterizing how the prototype θ-defensin, RTD-1, inhibits HPV16 (our model high-risk genotype). Similar to HD5, we found evidence that HPV viral inhibition by RTD-1 is facilitated directly through defensin-viral capsid interactions (20). This observation initially came from the combination of approaches taken that show infection inhibition is lost when serum is present during incubation or cells are pretreated with RTD-1. Additionally, we showed that dialyzing out unbound RTD-1 from RTD-1 + PsV combinations still results in significant infection reductions, further supporting this conclusion.

While both HD5 and RTD-1 interact directly with HPV virions, their inhibitory mechanisms differ. Alpha defensin HD5-mediated inhibition comes from the stabilization of the HPV viral capsid through charge-driven interactions with the C-terminal tail of the L1 major capsid protein and dimerized HD5. This directly prevents viral uncoating in endosomal compartments and redirects virion trafficking to the lysosome (33, 43). We show through our data that RTD-1 mediated inhibition of HPV is driven primarily through capsid clustering, which limits the capacity of virions to bind to the cell surface and prevents virion uptake. This mechanism of viral inhibition by θ-defensins has been documented before with IAV, where it was hypothesized that the defensins were able self-associate once bound to viral surfaces (23). Unlike the study with IAV, however, we found that the inherent cationic charge of the θ-defensin isoform had an impact on how well RTD inhibited HPV uptake and infection. This difference may potentially be explained by the affinity of θ-defensins to self-associate when bound to enveloped vs. non-enveloped viruses or the availability/quantity of interacting partners on the viral surface and their changes in affinity when interacting with θ-defensins. One such candidate for our study was viral-associated HSPGs.

Infectious uptake of HPV is a highly complex dance of virion associations with factors found on the cell surface and surrounding basement membrane. One of the key interactions that has been found is that binding of HPV virions to HSPG and growth factors can cause intracellular signaling prior to endocytosis of virions (36, 44). Given that the production of HPV PsV requires overnight maturation of a whole cell lysate, our purified PsV may have been pre-decorated with these factors. Because of this we utilized HPV PsVs treated with trypsin and heparinase in an effort to eliminate HSPG-decorated particles prior to purification. The HSPG-negative particles were more resistant to RTD-1-mediated inhibition, however the effect was not significant enough to suggest RTD-1 interactions with viral-associated HSPG are the primary drivers of capsid clustering. Importantly, we also found that RTD-1 is able to inhibit furin pre-cleaved HPV16 PsV. Furin pre-cleaved PsVs are able to bypass the initial HSPG binding interactions at the cell surface that are hypothesized to precede infectious uptake through a non-canonical, clatherin-independent, macropinocytosis-like mechanism that utilizes tetraspanins CD151 and CD63, integrins, growth factor receptors, the annexin A2 heterotetramer, actin, and obscurin-like protein 1 (reviewed in Mikulicic et al.) (36, 39, 45, 46). Given that it is still not known whether WT virions come furin pre-cleaved, uncleaved, or in a mixed population as seen in WT preps from organotypic raft cultures (47), it is reassuring to note that fpc PsV were just as susceptible to inhibition by RTD-1 when compared to our normal PsV preparations.

HPV16 is currently found in a majority of HPV+ head and neck cancer cases (48), however is not the only genotype associated with HPV-driven carcinogenesis. Because of this it was important to examine the cross-genotype inhibitory effects of RTD-1 on HPV18, and 31, which together with HPV16 make up the three most prominent genotypes associated with cervical cancer (49, 50). We found the same inhibitory capacity of RTD-1 on each genotype tested, making it is reasonable to assume that RTD-1 may be considered as a prophylactic measure against hrHPV infection with genotypes most commonly associated with HPV+ cancers. These findings open the door to considering the usages of θ-defensins as a way to reduce the infection and spread of HPV within the population.

Previous groups have proposed the application of human defensins as broad-spectrum antiviral agents that could be used to stop the transmission of viral and bacterial infections (20, 23). There are two significant limitations to this however: 1) the non-cyclic nature of α and β defensins makes them less stable and susceptible to degradation and 2) physiological concentrations of NaCl, Ca^2+^, and Mg^2+^ can modulate the anti-microbial activity of certain defensins (51–53). While we had shown in our data that excess divalent cations in solution (in the form of Ca^2+^) were able to prevent the viral aggregating effects of RTD-1, these concentrations were at 100 mM, which is not found physiologically (54). Given that a previous group has demonstrated that RTD-1 can withstand heating to 100°C for 30 min, is not toxic to cells *in vitro* or to mice in doses up to 160 mg/kg, does not break down in serum after a 72 h incubation, does not induce inflammation, and remains intact when stored at extreme pH (2.0), we propose that RTD-1 should be also be considered as a candidate for future development into a topical antiviral agent against hrHPV if it shows *in vivo* efficacy within the murine HPV vaginal challenge model (19, 55, 56). Application wise, it should be investigated whether RTD-1 could be added to lubricants currently used in packaged condoms to increase their ability to limit transmission or as a stand-alone product to prevent the spread of HPV and potentially other sexually transmitted infections in high-risk populations who are either unvaccinated or not eligible for prophylactic vaccination (57, 58). Furthermore, because θ-defensins are not naturally produced in humans it would be much easier to interpret efficacy as you would not have to contend with variable endogenous production of human defensins between study participants. An intriguing question, however, is whether topical application of θ-defensins would lead to dysbiosis of the microflora of mucosal sites as it is shown to have significant antimicrobial effects against *Escherichia coli, Staphylococcus aureus, and Candida albicans* through enhancement of host directed mechanisms (14, 59). Additionally, it would have to be determined whether naturally occurring mucosal proteins or semen would prevent anti-viral activities of RTD-1 and if this could be overcome by increasing concentration.

Future studies examining the interactions between HPV and θ-defensin family members should explore whether human retrocyclin isoforms or those occurring in olive baboons also have anti-viral efficacy, and whether RTD-1 viral aggregates show changes in opsonization rates by macrophages or dendritic cells (DC) (18). Previous work examining IAV aggregates formed by θ-defensin interactions showed enhanced uptake by macrophages and neutrophils, however it has yet to be explored as to whether DC antigen uptake and priming of anti-viral responses would be impacted (23, 60). Toward this goal, we examined whether the RTD-1 + HPV16 viral aggregates were immunogenic to Langerhans cells, the antigen presenting cells (APC) of the epithelium. Unfortunately, we found no differences in LC maturation or activation state when HPV16 + RTD-1 complexes were compared to WT HPV16 treated LC (unpublished data). While it would have been exciting to find that we could both inhibit viral transmission and overcome the tolerant phenotype LC taken on after exposure to HPV virions (61, 62), it appears as though tolerogenic interactions by HPV viral aggregates are maintained. Future studies looking to use RTD-1 mediated viral aggregates as an attempt to generate antiviral responses in APC populations other than LC could consider the addition of adjuvants that enhance humoral responses to bolster the production of neutralizing antibodies against the viral vector (63).

Overall, we have documented that θ-defensins from rhesus macaques can be utilized to inhibit hrHPV infection. Through an aggregation of virions that is contingent upon the inherent charge of the peptide, θ-defensins are able to prevent virions from binding to cell surfaces and subsequently reduce HPV uptake into target epithelial cells in a dose-dependent manner. This insight into the molecular interactions between θ-defensins and non-enveloped viruses may translate into a prophylactic strategy against hrHPV that would have a long shelf life and potentially informs future developments of anti-viral therapies against epithelial targeting pathogens through the usage of defensins.

## Data Availability Statement

The raw data supporting the conclusions of this article will be made available by the authors, without undue reservation.

## Author Contributions

Conceptualization and writing—original draft preparation: JS. Methodology: JS and WS. Formal analysis and investigation: JS, WS, and MG. Resources: JS, KL, DD, and WK. Writing—review and editing: JS, WS, MG, DF, RP, FV, DD, and WK. Supervision and project administration: DD and WK. Funding acquisition: WK. All authors have read and agree to the published version of the manuscript.

## Conflict of Interest

The authors declare that the research was conducted in the absence of any commercial or financial relationships that could be construed as a potential conflict of interest.

## Supplementary Material

Supplemental Figure 1 Schematic of the dialysis experiment from Figure 1D indicating how the different groups were created.

Supplemental Figure 2 Compared to WT HPV16 PsV, RTD-1 treated PsV show large virion clusters on specific cells in IF images. Immunofluorescence imaging of **(A)** untreated HPV16 PsV or **(B)** PsV incubated with 5.0 μg/mL RTD-1 prior to addition to cells.

Supplemental Video 1 Individual run of HPV16 VLP recorded on the NanoSight NS300.

Supplemental Video 2 Individual run of HPV16 VLP + 1 μg/mL RTD-1 recorded on the NanoSight NS300.

Supplemental Video 3 Individual run of HPV16 VLP + 2 μg/mL RTD-1 recorded on the NanoSight NS300.

Supplemental Video 4 Individual run of HPV16 VLP + 5 μg/mL RTD-1 recorded on the NanoSight NS300.

Supplemental Video 5 Individual run of RTD-1 @ 5 μg/mL recorded on the NanoSight NS300.
