# Supplementary figures and images for: Theta-Defensins Inhibit High-Risk Human Papillomavirus Infection Through Charge-Driven Capsid Clustering
Source: Front Immunol. 2020 Sep 25;11:561843. doi: 10.3389/fimmu.2020.561843 (PMC7586039; doi:10.3389/fimmu.2020.561843)

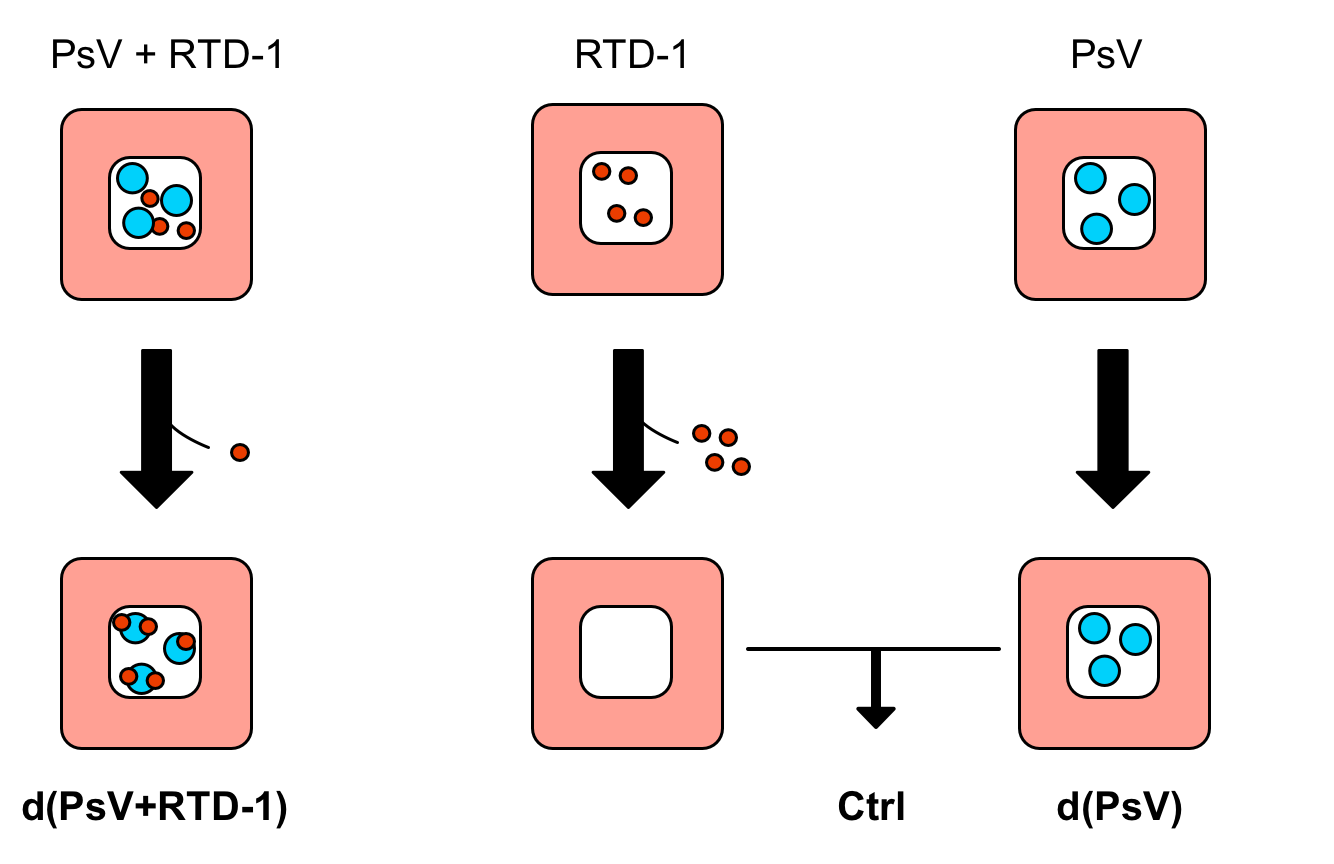

Supplement: Supplemental Figure 1 — Schematic of the dialysis experiment from Figure 1D indicating how the different groups were created. [file Image_1.TIFF]

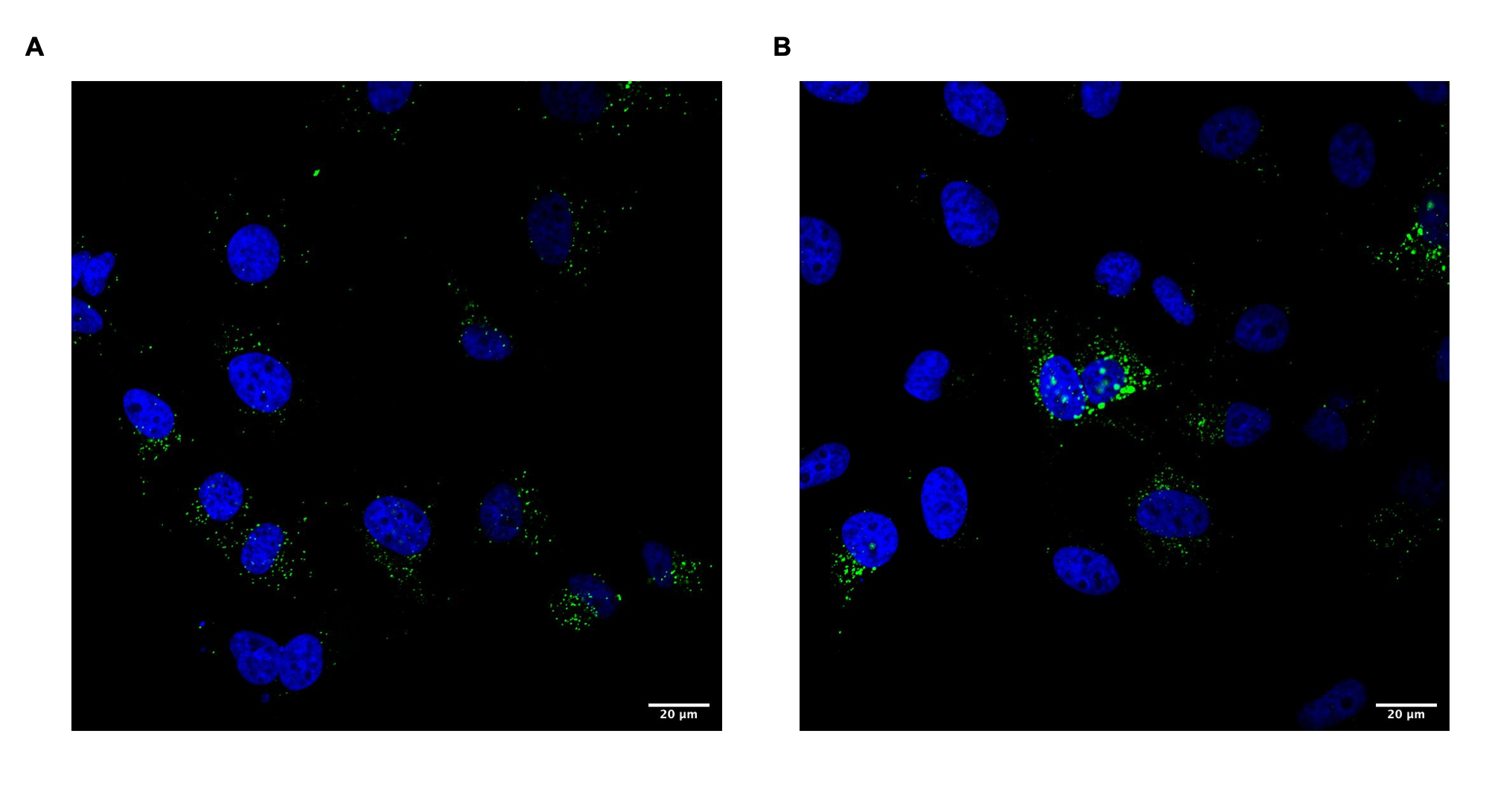

Supplement: Supplemental Figure 2 — Compared to WT HPV16 PsV, RTD-1 treated PsV show large virion clusters on specific cells in IF images. Immunofluorescence imaging of (A) untreated HPV16 PsV or (B) PsV incubated with 5.0 μg/mL RTD-1 prior to addition to cells. [file Image_2.TIFF]
